# Snacktivity™ to Promote Physical Activity in Primary Care, Community Health and Public Health Settings: A Feasibility Randomised Controlled Trial

**DOI:** 10.1007/s12529-025-10352-3

**Published:** 2025-02-24

**Authors:** Amanda J. Daley, Ryan A. Griffin, James P. Sanders, Kajal Gokal, Natalie Ives, Magdalena Skrybant, Helen M. Parretti, Charlotte L. Edwardson, Stuart J. H. Biddle, Kate Jolly, Colin J. Greaves, Sheila M. Greenfield, Ralph Maddison, Dale W. Esliger, Lauren B. Sherar, Emma Frew, Nanette Mutrie, Ben Maylor, Tom Yates, Sarah Tearne, Catherine A. Moakes

**Affiliations:** 1 Centre for Lifestyle Medicine and Behaviour, School of Sport, Exercise and Health Sciences, Loughborough University, Loughborough, UK; 2 Birmingham Clinical Trials Unit, College of Medicine and Health, University of Birmingham, Birmingham, UK; 3 Department of Applied Health Sciences, College of Medicine and Health, University of Birmingham, Birmingham, UK; 4 Norwich Medical School, Faculty of Medicine and Health, University of East Anglia, Norwich, UK; 5 University Hospitals of Leicester NHS Trust, College of Life Sciences, University of Leicester and NIHR Leicester Biomedical Research Centre, Leicester, UK; 6 University of Southern Queensland, Springfield, Australia; 7 Faculty of Sport & Health Sciences, University of Jyväskylä, Jyväskylä, Finland; 8 School of Sport, Exercise and Rehabilitation Sciences, University of Birmingham, Birmingham, UK; 9 Institute for Physical Activity and Nutrition, Deakin University, Melbourne, Australia; 10 School of Sport, Exercise and Health Sciences, Loughborough University, Loughborough, UK; 11 NIHR Leicester Biomedical Research Centre, Leicester, UK; 12 Health Economics Unit, Department of Applied Health Sciences, College of Medicine and Health, University of Birmingham, Birmingham, UK; 13 Physical Activity for Health Research Centre, University of Edinburgh, Edinburgh, UK

**Keywords:** Physical activity, Snacktivity™, Health, Randomised feasibility trial, Short bouts

## Abstract

**Background:**

A novel ‘whole day’ approach that could motivate the public to be more physically active is Snacktivity™. The Snacktivity™ approach encourages individuals to accumulate 150 min of physical activity in short 2–5-min ‘snacks’ of moderate-vigorous intensity physical activity (MVPA) throughout the day/week.

**Method:**

A randomised controlled trial to assess the feasibility/acceptability of a Snacktivity™ intervention and trial processes was conducted. The trial aimed to recruit 80 physically inactive adults from healthcare services and via social media. Participants were randomised to the Snacktivity™ intervention or usual care and followed up at 12 weeks. The intervention was predominately delivered by health professionals within consultations. Assessment of whether the Snacktivity™ intervention and trial methods were acceptable to participants, adherence to Snacktivity™ (assessed by Fitbit) and physical activity (assessed by accelerometer), and retention were considered according to traffic light stop-go progression criteria (green-amber-red).

**Results:**

Seventy-two participants (*n* = 37 Snacktivity™ intervention; *n* = 35 usual care) were recruited across 14 months (72/80, 90%, (green) 95% CI: 83% to 97%). Snacktivity™ adherence was achieved in 12/37 participants (32%, (red) 95% CI: 17% to 48%). Physical activity adherence was achieved in 17/37 participants (46%, (amber) 95% CI: 30% to 62%). Seven participants (10%, (green) 95% CI: 3% to 17%) withdrew from follow-up and 25/72 (35%, (amber) 95% CI: 24% to 46%) had no accelerometer data at follow-up (retention).

**Conclusion:**

The Snacktivity™ intervention may be feasible and acceptable to implement. Findings can inform subsequent research that seeks to investigate whether Snacktivity™ based approaches are effective in promoting physical activity in the population.

**Trial Registration:**

ISRCTN: 64851242. Registration date: 31/01/21.

**Supplementary Information:**

The online version contains supplementary material available at 10.1007/s12529-025-10352-3.

## Background

Many people do not participate regularly in physical activity, which may negatively affect their health and well-being [1, 2]. In the past, physical activity guidelines have focused on promoting the accumulation of at least 150 min of moderate intensity physical activity per week, or 75 min of vigorous intensity, or a combination of both (MVPA) [3]. Guidance now recognises the contribution to health that participation in short bouts of physical activity can have, and that any amount of physical activity is better than none [4, 5]. This change to the guidance has provided opportunities to develop interventions that promote short(er) bouts of physical activity to the public. Guidance also advises that adults should complete muscle strengthening physical activities on at least 2 days per week [4]. However, few adults (< 20%) achieve this goal for strength-based physical activity, despite strong evidence that this form of activity is effective in reducing the risk of multiple health conditions and all-cause mortality [6–8]. This low participation rate highlights the need to find ways to engage the public in these types of physical activity.

Support for the notion that short bouts of accumulated physical activity might improve health can be drawn from findings of systematic reviews and several experimental studies that have shown no differences between continuous and accumulated exercise bouts on metabolic health outcomes immediately post exercise [9–13]. However, evidence from real-world randomised controlled trials (RCTs) is required to develop knowledge about whether the accumulated approach to physical activity is acceptable and feasible for the public within daily life over time. Moreover, guideline committees have called for more longitudinal research to test the health benefits of physical activity accumulated in bouts less than 10 min.

## Snacktivity™ to Promote Physical Activity

Given that health agencies around the world have removed the necessity to complete physical activity in bouts lasting ≥ 10 min to contribute towards achieving the guidelines [4, 5], an approach that could encourage and motivate the public to be more physically active is a concept we have termed Snacktivity™ [14]. Snacktivity™ focuses on encouraging participation in brief, but frequent, ‘snack size’ bouts of MVPA and muscle/strength-based physical activity, which is accumulated across the day/week to meet the guidance. An activity snack typically lasts between 2 and 5 min and can be incidental or planned. Examples of activity snacks include brisk ‘walk and talk’ conversations, using stairs instead of the lift, calf raises when brushing your teeth and squats while waiting for the kettle to boil. Snacktivity™ aims to combine behaviours and concepts to translate the promotion of physical activity into a format that is novel and motivating to the public, regardless of ability, socio-economic status or the availability of equipment. Moreover, the potential convenience of accumulating Snacktivity™ through activities of daily living makes it accessible to almost everyone.

A common reason for inactivity is a perceived lack of time, and Snacktivity™ provides an opportunity to address this barrier, through promoting short and time efficient bouts of physical activity [15]. Snacktivity™ does not require any planning or equipment, it is not weather dependent and may be perceived by the public as appealing and feasible to achieve [16]. The Snacktivity™ approach may help to develop individuals’ confidence to be active by encouraging them to ‘start small’ [14]. Simple actions are more likely to become habitual more quickly than complex ones, suggesting that integration of activity snacks within everyday routines may be easier to initiate, and maintain [17].

Adults spend approximately 60–70% of their waking hours sedentary which can negatively impact health [18–20]. Guidelines now include recommendations about reducing time spent sedentary. By design, Snacktivity™ naturally and conveniently encourages breaking up sitting time during the day, through participation in regular activity snacks. This means that Snacktivity has the ability to impact two health behaviours simultaneously, potentially contributing towards making the approach cost-effective.

Our earlier observational and qualitative work focused on developing the Snacktivity™ concept, which has shown that a short bout-based approach to promoting physical activity is viewed positively by the public [16, 21, 22]. For example, it has been found that the public like the Snacktivity™ concept and that the approach feels more manageable to achieve than longer bouts of physical activity [16, 21, 22]. Analogous concepts to Snacktivity™ include ‘snackercising’ and vigorous/moderate intermittent lifestyle physical activity (VILPA and MV-ILPA) [23–25], which have also been supported by the public [23].

The Snacktivity™ message is one way the translation of guidelines for physical activity could be achieved. However, it is not an approach that has been highlighted to the public, in part, because of a lack of evidence from RCTs that the approach impacts long(er)-term health. Specifically, no RCT has investigated whether a Snacktivity™ intervention increases participation in MVPA and improves health outcomes over time. Following completion of several Snacktivity™ intervention development studies [16, 22, 23], the primary aim of the Snacktivity™ programme is to evaluate the clinical effectiveness and cost-effectiveness of a Snacktivity™ intervention for increasing physical activity in a large multi-centre RCT. However, research needs to first assess the feasibility and acceptability of the approach, to inform a future phase III trial [26]. There are also uncertainties regarding the best methods and rates of recruitment, the likely level of adherence to a Snacktivity™ intervention and participant retention. This study aims to address these questions.

## Methods

### Trial Design and Setting

A two-arm, multi-centre, individually randomised controlled feasibility trial was conducted across the Midlands, UK, with a target to recruit and allocate 80 participants to either the Snacktivity™ intervention group or usual care. Recruitment was through the National Health Service (NHS), public health, primary care, and community settings, and through social media adverts. Follow-up took place 12 weeks following randomisation. Two qualitative studies, one with participants who received the intervention and another with healthcare providers (HCPs) who delivered the Snacktivity™ intervention to participants, were embedded in the trial (not reported here). A detailed protocol for this trial has been published [26]. The trial is reported in line with the CONSORT checklist for feasibility trials [27].

### Recruitment of Participants, Eligibility and Consent

Participating general practices and NHS Trusts searched their electronic patient record systems to identify patients who were ≥ 18 years and had a healthcare consultation booked during the recruitment phase of the trial. These patients were sent a consultation appointment letter (or reminder for the upcoming appointment), along with the study information pack which contained the trial invitation letter, participant information sheet, expression of interest form (EOI), and the General Practice Physical Activity Questionnaire (GPPAQ) for screening physical activity status [28]. Some practices sent their patients a text message link to access these documents online. Potential participants provided consent for all steps of the screening process described. HCPs could also raise the study with patients during routine consultations and those interested in taking part were asked to complete the EOI form and GPPAQ and return these to the research team. Participants recruited via these routes had the Snacktivity™ intervention or usual care delivered by their healthcare provider. Potential participants were also invited to take part via community settings, including social media, and these participants were either sent the study information pack as detailed above by post or asked to complete the study documents electronically using the study invitation link. These participants received the Snacktivity™ intervention or usual care from a researcher.

Those identified as inactive, moderately inactive/active according to the GPPAQ (based on questions 1, 2a and 2b) were contacted by telephone by a member of the research team to complete eligibility screening. Thereafter, to be eligible, people needed to provide informed consent, aged ≥ 18 years, own a mobile phone capable of hosting apps (Apple or Android) and agree that their HCP could be notified of their involvement in the study (if applicable). Those unable to understand English sufficiently to complete the trial assessments and women known to be pregnant or breast feeding were ineligible. Once eligibility screening was completed, a study visit with participants was organised to obtain full informed consent for the trial and to collect baseline data. Where study visits were not possible, participants were asked to provide written informed consent either online/email or by post.

### Randomisation and Blinding

Once consent had been obtained and all baseline data collected (see below), participants were individually randomised (1:1 ratio) to either the Snacktivity™ intervention group or usual care. Randomisation was performed via a secure web-based service provided by the Birmingham Clinical Trials Unit. A minimisation algorithm was used to ensure balance in the treatment allocation for the following variables: route of recruitment (primary care, community health service, other); age (18–45, ≥ 46 years); and gender (male, female). A random element was included in the minimisation algorithm to ensure allocation concealment. The trial treatment allocations were sent directly to participants’ HCPs prior to their consultation or the researcher responsible for delivering the intervention or usual care.

### Blinding

Participants could not be blinded to the purpose of the trial. It was also not possible to always blind data collectors. We do not believe this introduced bias, as the aim of the trial was to assess the feasibility and acceptability of undertaking a large multi-centre phase III RCT, where the planned primary outcome of device-measured physical activity would not be affected by knowledge of group allocation. Furthermore, data relating to the feasibility outcomes were not collected during the follow-up visit, with only data relating to secondary outcomes collected by researchers at this visit.

### The Snacktivity™ Intervention

The intervention promoted participation in Snacktivity™, the usefulness of Snacktivity™ as an approach to behaviour change, encouraged regular self-monitoring of Snacktivity™ to achieve sustained behaviour, goal setting for daily Snacktivity™, and action planning and implementation strategies for Snacktivity™. The Snacktivity™ intervention is based on self-regulation theory and the habit formation model [29, 30]. Research has shown self-regulation/self-monitoring to be an effective foundation strategy for health behaviour change [31–33]. Self-monitoring of Snacktivity™ may act as a reward for individuals who increase their physical activity behaviour, who are then provided with positive feedback from the monitoring process, thereby enhancing their motivation and reducing the potential for relapse. Frequent monitoring and reflection of Snacktivity™ progress may also improve self-efficacy for participation in both short and longer bouts of physical activity.

The Snacktivity™ intervention involved two principal components, a Snacktivity™ consultation and signposting to using the Snacktivity™ support technology (mobile phone app called SnackApp™ linked to a Fitbit Versa 2 device) (Fitbit Inc, Google LLC, San Francisco, USA). The Making Every Contact Count (MECC) initiative in England seeks to embed conversations about health behaviour change into routine consultations between patients and health professionals [34]. Consistent with MECC, participants recruited through health services received the Snacktivity™ intervention from their HCP during a routine consultation. Participants recruited via social media received their Snacktivity™ consultation from a researcher. The main focus of the consultation was on raising awareness of, and encouraging Snacktivity™, and promotion of the intervention technology to support behaviour change and sustained engagement in Snacktivity™. The intervention period was 12 weeks.

Participants randomised to the Snacktivity™ intervention were advised to achieve their physical activity through activity snacks, 2–5-min bouts of MVPA throughout the day, to accumulate ≥ 150 min of MVPA weekly. Consistent with guidance, participation in muscle strength-based activity was also encouraged.

The Snacktivity™ intervention aimed to promote participation in activity ‘snacks’, and by encouraging regular self-monitoring to achieve sustained participation in Snacktivity™, goal setting, action planning and implementation strategies for activity snacking. The Snacktivity™ picture board that illustrated examples of aerobic and strength/resistance-based activity snacks was also given to participants during their consultation or emailed in advance for participants who received their consultation remotely (online or telephone). See Electronic Supplementary Material 1 for examples of activity snacks. Participants were encouraged to select activity snacks based on their own preferences and Snacktivity™ is designed to be completed at home and work, or any other setting that participants select. As part of the intervention consultation, participants were informed of who to contact in the event of issues with the Fitbit and/or SnackApp™. Setup and charging instructions for the Fitbit and SnackApp™ detailing that there was a dedicated helpline for any issues with the technology were also sent to participants.

**Fig. 1:**
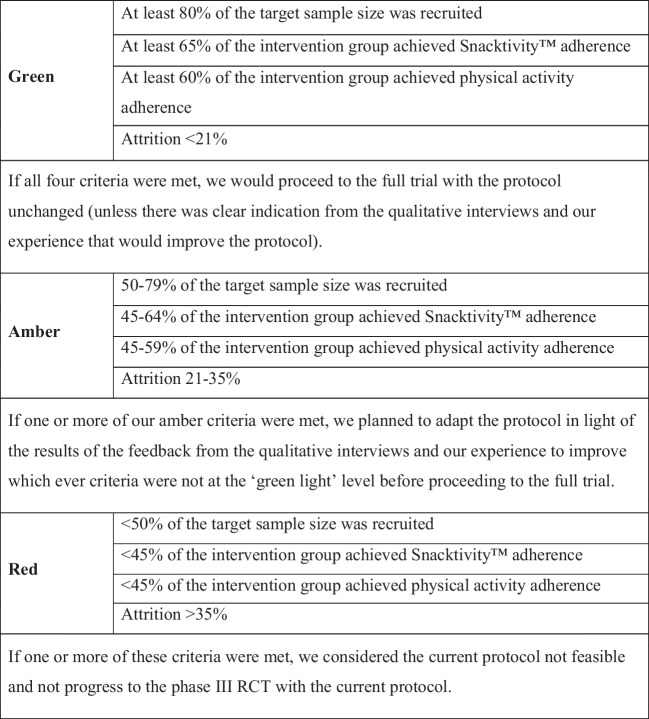
Traffic light stop–go criteria

The purpose of the Fitbit device and SnackApp™ for facilitating self-monitoring of activity snacks was discussed with participants in the consultations and use of this bespoke Snacktivity™ technology for this purpose was encouraged. The SnackApp™ included over 50 different examples/ideas of activity snacks that participants could complete [35]. To facilitate habit formation and action planning, the SnackApp™ generated regular reminders and notifications for participants to engage in Snacktivity™. Self-monitoring may be particularly relevant for developing Snacktivity™ habits because it may be more difficult for people to easily recall how many activity snacks they have achieved each day/week. The content and design of the SnackApp™ are based on previous work [35]. Participants were given free access to the SnackApp™ and Fitbit after their intervention consultation. The Fitbit device provided data to participants on the number of activity snacks, ‘active minutes’ (i.e. MVPA) and steps they were achieving each day using a bespoke study SnackApp™ clockface on the device (Electronic Supplementary: Fig. 1). Participants were asked to wear the Fitbit throughout the 12*-*week intervention period.

### Training of HCPs/Researchers to Deliver the Snacktivity™ Intervention

Those delivering the intervention were trained by the research team to deliver the Snacktivity™ intervention following a standard protocol and intervention checklist. The research team developed a 30-min media-based training module that could be delivered face-to-face or remotely (see protocol publication for details [26]). Consultations were audio-recorded to assess fidelity against the intervention checklist, and this is reported in a separate publication [36]. Details regarding the development of the intervention against an Intervention Mapping framework have been published previously [35].

### Comparator Group

During their healthcare or telephone consultation with a researcher, the usual care group received the current guidance for physical activity in the UK and were advised to work towards the accumulation of at least 150 min of MVPA per week. Participants also received a leaflet that promoted physical activity (in person or by email). The usual care consultations were audio-recorded to assess for intervention contamination.

### Primary Outcome and Progression Criteria for Phase III Trial and Stop–Go Criteria

The primary outcome was the feasibility and acceptability of a subsequent phase III RCT according to pre-specified progression criteria (see Fig. 1). The primary purpose was to investigate whether the Snacktivity™ intervention and trial were appealing to participants (assessed by the recruitment rate) and if the intervention and the evaluation methods were acceptable to participants (measured by Snacktivity™ and physical activity adherence and trial retention rates). The progression criteria were considered in the context of a traffic light system to indicate if the trial should proceed to an effectiveness trial; stop/red; intractable issues that cannot be remedied; amend/amber: remediable issues, proceeding with caution; and continue/green: no concerning issues that threaten the success of the trial [37].

As detailed in Fig. 1, four pre-specified progression criteria were used to guide the assessment of whether to continue to a phase III RCT: (1) recruitment (the number/percentage of people randomised against the recruitment target of 80 participants over 5 months); (2) Snacktivity*™* adherence (the number of physical activity snacks achieved (defined as minimum of four bouts of MVPA lasting ≥ 2 min on average each day over 12 weeks) assessed by the Fitbit device (Snacktivity*™* group only)); (3) physical activity adherence (the proportion of participants who accumulated a total weekly average of ≥ 105 min of MVPA (Snacktivity*™* group only)) as assessed by wrist-worn accelerometry; and (4) attrition (withdrawal from the trial and/or no follow-up data available). A pragmatic decision was made for the selection of ≥ 105 min of MVPA to determine physical activity adherence because inactive participants may have low confidence, and who were being encouraged to achieve the guidance over time. This is also in line with guidance that states every minute counts. Thresholds for these progression criteria are detailed in Fig. 1.

### Secondary and Process Outcomes

Data were collected on outcomes that were expected to be collected in the phase III trial (Electronic Supplementary Material 2: Table 1). This feasibility trial was not statistically powered to detect meaningful differences in outcomes, but collecting these data provided the opportunity to ensure there were no issues with data collection before a phase III trial. At baseline and follow-up, accelerometer measured minutes of participation in MVPA, total physical activity, light physical activity, and sedentary and sleep time. Self-reported wake and sleep time logs were also completed on the days the accelerometer was worn. A checklist of activity snacks was completed at follow-up in the Snacktivity™ intervention group. Participants’ experiences of the trial (all participants) and the intervention (Snacktivity™ group) were assessed through a series of single-item questions at follow-up. We wished to assess the recruitment and randomisation processes, measure the extent of any intervention contamination, and use data collected in the feasibility trial to review the sample size assumptions for the phase III trial. Data regarding adverse events were collected as per the protocol [26].

Other data collected at baseline and follow-up in both groups were lower limb muscle strength (Takei dynamometer in squat position), weight (kg), body mass index (BMI), waist circumference (cm), blood pressure, self-reported sedentary behaviours (Workforce Sitting Questionnaire (WSQ)) [38], sedentary behaviour items from the International Physical Activity Questionnaire (IPAQ) [39] and anxiety/depression (Hospital Anxiety and Depression Scale) [40]. Participants also completed the Physical Activity Enjoyment Scale [41] and Exercise Self-efficacy Questionnaire [42] and the Self-Report Habit Index (for Snacktivity™) [43] but this data is not reported. Items planned to be used to assess healthcare resource use and productivity were piloted for use in the phase III trial but not reported. The data that support the findings of this study are available on request from the corresponding author. The data are not publicly available due to the data containing information that could compromise the privacy of participants in the study. The data that support the findings of this study are available on request from the corresponding author.

### Data Collection

Study visits for the collection of measured data were conducted in participants’ homes, a community venue or general practice by a researcher. Participants were asked to wear a blinded research-grade Axivity accelerometer on their non-dominant wrist for seven consecutive days (Axivity AX3; Axivity, Newcastle, UK) and to complete the study questionnaires (online link or posted copy) before the baseline study visit. Data was collected again as per baseline at 12-week follow-up (Electronic Supplementary Material: Table 1 details the assessments for data collection). In the Snacktivity™ intervention group, the number of activity snacks completed, steps, distance, calories, physical activity minutes (active minutes), inactive time (proxy for sedentary time), sleep and awake time and wear time per day/week were derived from the SnackApp/Fitbit. Participants received a £20 voucher at follow-up for completing the study.

### Sample Size and Data Analysis

The trial aimed to recruit 80 participants over 5 months. The sample size was determined following recommendations for appropriate sample sizes to be used in feasibility trials [44]. A sample size of 80 participants (*n* = 40 intervention) would allow us to estimate a Snacktivity™ adherence rate of 65% to within a 95% confidence interval (CI) of ± 14.8%; a physical activity (MVPA) adherence rate of 60% to within a 95% CI of ± 15.2%; and an attrition rate of 20% to within a 95% CI of ± 8.8% (all participants). Based on a response rate of between 1 and 2%, 4000 to 5000 people needed to be invited to achieve the required target of 80 participants.

A statistical analysis plan was agreed before any analysis was undertaken. Outcomes were analysed using the intention-to-treat principle (where data were reported by group). Data analysis was predominately descriptive and focused on estimating confidence intervals (CIs). Hypothesis testing was not performed, and *p*-values are not presented. In relation to the trial progression criteria, recruitment and attrition rates were analysed by pooling the two randomised groups, whilst adherence (Snacktivity™ and physical activity) rates were calculated for the Snacktivity*™* group only. For continuous secondary outcome measures, means and standard deviations were reported for each randomised group alongside mean differences (with 95% CIs) estimated using a linear regression model adjusted for age, gender, route of recruitment and baseline value. Sedentary behaviours measured using the IPAQ and the WSQ were presented using medians and interquartile ranges and unadjusted differences in medians (with 95% CIs) were produced for total sitting time using bootstrapping methods. All participants reported their experiences of the trial (single items) and the Snacktivity™ group also reported their experiences of the intervention and these data are reported descriptively. All analyses were conducted in SAS (version 9.4) or Stata (version 18.0).

### Public and Patient Involvement (PPI)

The Snacktivity™ Public Advisory Group (PAG), comprised of 10 members from a range of backgrounds and with different experiences of physical activity, were key partners in the design and delivery of the trial. The group were involved in co-design activities, including co-developing the SnackApp throughout its development, developing the recruitment strategy, shaping the content and format of participant information sheets and consent form, and providing input to specific resources used as part of the intervention (e.g. a picture board showing examples of short bouts of physical activity). Two public contributors from the PAG provided public insights on the monthly Trial Management Group meetings. A public contributor from the PAG also attended the Trial Steering Committee meetings throughout the trial.

## Results

### Recruitment of Participants, Baseline Characteristics and Participant Flow

**Fig. 2:**
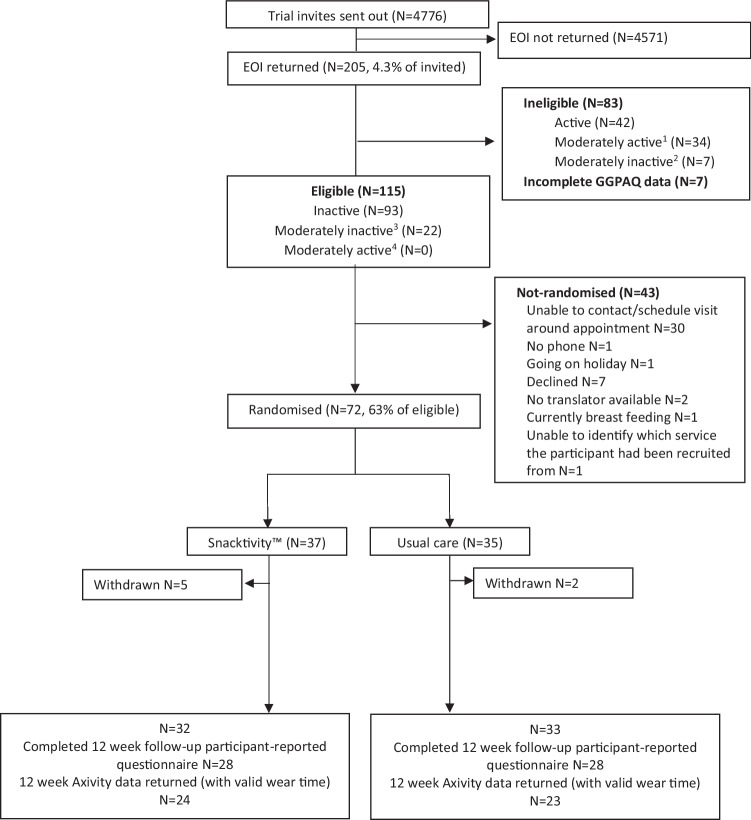
Participant flow

In total, 4776 invitations were sent and 205 EOIs were returned (4.3%), and of these, 115 (56%) participants were considered eligible and 72 were randomised (intervention, *n* = 37; usual care, *n* = 35). Of those randomised, 78% (*n* = 56) completed the self-reported outcomes and 65% (*n* = 47) provided accelerometer data at follow-up (Fig. 2 for participant flow). Most participants were recruited via general practices (*n* = 34), with the remaining recruited through community health services (*n* = 24; from across physiotherapy *n* = 12; dentistry *n* = 9; podiatry *n* = 2; dietetics *n* = 1), an NHS mental health service (*n* = 3) (patients recruited via health services, *n* = 61) and social media/adverts (*n* = 11). See Table 1 for participants characteristics. The first participant was recruited in September 2021 and follow-up was completed in November 2022.

**Table 1:** Baseline characteristics

Participant characteristic	Snacktivity™ *N* = 37	Usual care *N* = 35
Recruiting service/route*—*N* (%)		
Primary care	16 (43)	18 (51)
Community health service	13 (35)	11 (32)
Other^1^	8 (22)	6 (17)
Age at randomisation (years)*—*N* (%)		
18–45	10 (27)	7 (20)
≥ 46	27 (73)	28 (80)
Mean (SD, *N*)	53.6 (12.5, 37)	55.1 (13.8, 35)
Gender*—*N* (%)		
Male	7 (19)	7 (20)
Female	29 (78)	28 (80)
Missing/prefer not to say	1 (3)	0
GPPAQ group^2^—*N* (%)		
Moderately inactive	9 (24)	7 (20)
Inactive	28 (76)	28 (80)
Ethnicity—*N* (%)		
English/Welsh/Scottish/Northern Irish/British	28 (75)	24 (69)
Irish	0 (-)	1 (3)
Other white background^3^	1 (3)	2 (6)
White and Asian	1 (3)	0 (-)
Any other Mixed/Multiple ethnic background^3^	0 (-)	0 (-)
Indian	2 (5)	3 (8)
Pakistani	2 (5)	3 (8)
Chinese	1 (3)	0 (-)
Any other Asian background^3^	1 (3)	0 (-)
Caribbean/African	0 (-)	2 (6)
Any other ethnic group^3^	1 (3)	0 (-)
Marital status—*N* (%)		
Single, never married	3 (9)	7 (20)
Married or domestic partnership	25 (71)	24 (71)
Separated, divorced, widowed	7 (20)	3 (9)
Prefer not to say	2	1
Current employment status—*N* (%)		
Employed full time (35 or more hours a week)	12 (32)	12 (34)
Employed part time (less than 35 h per week)	7 (19)	5 (14)
Unemployed	5 (14)	5 (14)
Student	0 (-)	0 (-)
Retired	10 (27)	10 (29)
Self-employed	1 (3)	2 (6)
Unable to work	2 (5)	1 (3)
Number of cigarettes smoked daily—*N* (%)		
None	32 (86)	34 (97)
≤ 5	0 (-)	0 (-)
6–10	3 (8)	1 (3)
11–15	1 (3)	0 (-)
16–19	0 (-)	0 (-)
≥ 20	1 (3)	0 (-)
Alcohol consumption in the last week (yes/no)—*N* (%)	21 (57)	18 (51)
Mobility—*N* (%)		
Fully ambulant without walking aid	34 (92)	33 (94)
Ambulant with walking aid	3 (8)	2 (6)
Medical history—*N* (%)		
Cancer	3 (8)	2 (6)
Type 1 diabetes	0 (-)	1 (3)
Type 2 diabetes	3 (8)	1 (3)
High cholesterol	4 (11)	8 (23)
High blood pressure (hypertension)	10 (27)	3 (9)
Heart disease, heart attack, angina, aneurysm	0 (-)	1 (3)
Stroke	0 (-)	0 (-)
Depression or anxiety	12 (32)	12 (34)
Dementia or Alzheimer’s disease	0 (-)	0 (-)
Osteoporosis	0 (-)	1 (3)
Obesity	3 (8)	4 (11)
Sarcopenia	0 (-)	0 (-)
COPD/emphysema	1 (3)	(-)
Asthma	5 (14)	5 (14)
Kidney disease	0 (-)	1 (3)
Back pain resulting in time off work	7 (19)	5 (14)
Rheumatoid arthritis	0 (-)	3 (9)
Osteoarthritis	4 (11)	4 (11)
Neurological condition (e.g. epilepsy, ME or MS)	1 (3)	0 (-)
COVID-19	7 (19)	9 (26)
Foot/ankle problem affecting mobility	1 (3)	6 (17)
Other^4^	4 (11)	5 (14)
Height (cm)—mean (SD, *N*)	166.4 (10.4, 37)	163.1 (9.6, 35)
Weight (kg)—mean (SD, *N*)	81.4 (20.9, 37)	83.4 (27.6, 35)
BMI (kg/m^2^)—mean (SD, *N*)	29.4 (7.2, 37)	31.2 (9.5, 35)
Waist circumference (cm)—mean (SD, *N*)	97.3 (16.4, 37)	98.8 (15.7, 35)
Lower limb muscle strength (kg)—mean (SD, *N*)	48.1 (23.9, 27)	45.5 (19.6, 28)
Systolic blood pressure (mmHg)—mean (SD, *N*)	124.4 (18.6, 37)	129.0 (20.4, 34)
Diastolic blood pressure (mmHg)—mean (SD, *N*)	77.9 (9.8, 37)	78.8 (11.3, 34)

Abbreviations:* BMI* body mass index, *cm* centimetres, *COPD* chronic obstructive pulmonary disease, *GGPAQ* General Practice Physical Activity Questionnaire, *IQR* interquartile range, *kg* kilogrammes, *m* metres, *mmHg* millimetres of mercury, *ME* myalgic encephalomyelitis, *MS* multiple sclerosis, *N* number of observations *SD* standard deviation

^*^Minimisation variable

^1^Other services/routes: Adult mental health service and social media

^2^Inactive participants recruited via protocol versions ≥ 1.0. Moderately inactive participants recruited via protocol versions ≥ 6.0. Moderately active participants recruited via protocol version 7.0

^3^Other ethnicities include Polish, Persian, Danish, Russian and assumed Caucasian Anglo-Saxon

^4^Other diseases or conditions include fibromyalgia, irritable bowel syndrome (IBS), Raynauds, polycystic ovarian syndrome, hyperthyroidism, Meniere’s disease, ventricular ectopic beats, migraines, prolapsed lumbar disc, pre-diabetes and sleep apnoea

### Feasibility Traffic Light Assessment

The green criterion for participant recruitment was achieved (72/80, 90%, 95% CI: 83% to 97%), but over a 14-month recruitment period, not 5 months as planned. Concerning adherence to Snacktivity™, two different scenarios are presented. In scenario 1, we included all the Fitbit data and assumed activity levels on days with no Fitbit wear time were the same as activity levels on days with wear time. Under this assumption, the green stop–go criterion was achieved. In scenario 2, only participants who had data available for at least 5/7 days each week for at least 9/12 intervention weeks were included. Under this assumption, the red criterion was met. All data for these results are presented in Table 2. Snacktivity™ data across week and weekend days are summarised in Table 3, where the intervention group completed on average 10.1 activity snacks on weekdays and 9.0 on weekend days. Accelerometer-measured physical activity adherence met the amber criteria under two scenarios. In scenario 1, we included all participants with at least 1 day of valid wear at follow-up. In scenario 2, we included participants with at least 4 days of valid wear at follow-up. Attrition met the amber or green criteria, depending on the assumptions that are applied (Table 2).

**Table 2:** Results for the stop–go criteria

Criteria—*N*/*N* (%)	Estimate [95% CI]	Target met
Recruitment rate*	72/80 (90%) [83% to 97%]	Green [95% CI: Green to Green]
Snacktivity™ adherence rate^1^ (scenario 1—including all data and average over available data only)		
Adherent	25 (68%) [52% to 83%]	Green [95% CI: Amber to Green]
Non-adherent	2 (5%)	
Missing**	10 (27%)	
Snacktivity™ adherence rate^1^ (scenario 2—only including participants who had data available for at least 5 of 7 days each week for at least 9 of 12 weeks)		
Adherent	12 (32%) [17% to 48%]	**Red** [95% CI: Red to Amber]
Non-adherent	0 (-)	
Insufficient wear time	15 (41%)	
Missing**	10 (27%)	
Physical activity adherence^1^ (scenario 1: Including all participants with ≥ 1 day of valid wear)		
Adherent	20 (54%) [38% to 70%]	**Amber** [95% CI: Red to Green]
Non-adherent	4 (11%)	
Missing***	13 (35%)	
Physical activity adherence^1^ (scenario 2: Including all participants with ≥ 4 days of valid wear)		
Adherent (≥ 4 days of valid wear)	17 (46%) [30% to 62%]	**Amber** [95% CI: Red to Green]
Non-adherent (≥ 4 days of valid wear)	3 (8%)	
< 4 days of valid wear	4 (11%)	
Missing***	13 (35%)	
Attrition rate (including participants who withdrew from follow-up only)	7/72 (10%) [3% to 17%]	**Green** [95% CI: Green to Green]
Attrition rate (including participants withdrew from follow-up and who had no Axivity data)	25/72 (35%) [24% to 46%]	**Amber** [95% CI: Amber to Red]

^*^Recruitment in 14 months

^**^No Fitbit data (includes participants who had withdrawn (*N* = 5) and those who returned Fitbit devices with no valid wear time (*N* = 5))

***No axivity data (includes participants who have withdrawn (N=5), those who did not return their watches (N=2) and those who returned their watches with no valid wear time (N=6))

^1^In the Snacktivity™ arm only (*N* = 37)

**Table 3:** Fitbit physical activity data (Snacktivity™ intervention group)

	Weekday	Weekend	Total
Number of minutes of wear time/day			
Mean (SD, *N*)	1041.1 (255.3, 27)	1014.9 (323.9, 27)	1030.3 (263.9, 27)
Minimum–Maximum	418.1–1327.2	289.8–1388.8	402.7–1332.3
Number of steps/day			
Mean (SD, *N*)	8816.1 (3666.2, 27)	8873.2 (3978.7, 27)	8807.4 (3606.3, 27)
Minimum–Maximum	3714.5–17,589.8	1342.0–19,868.3	3764.7–17,001.3
Number of active minutes/day			
Mean (SD, *N*)	93.5 (54.7, 27)	93.3 (54.2, 27)	93.3 (53.2, 27)
Minimum–Maximum	15.0–210.6	16.0–246.1	15.0–221.4
Number of inactive minutes/day (proxy for sedentary time)			
Mean (SD, *N*)	706.1 (146.0, 27)	665.9 (212.9, 27)	691.8 (144.8, 27)
Minimum–Maximum	383.4–1024.6	255.0–1171.0	370.1–1037.6
Number of minutes of sleep time/day			
Mean (SD, *N*)	241.5 (210.5, 27)	255.7 (213.4, 27)	245.2 (209.9, 27)
Minimum–Maximum	0–519.0	0–535.1	0–524.1
Number of activity snacks (2–5-min bouts)/day			
Mean (SD, *N*)	10.1 (5.5, 27)	9.0 (5.1, 27)	9.8 (5.3, 27)
Minimum–Maximum	1.6–21.6	1.0–23.3	2.1–22.2
Number of activity bouts (> 5-min bouts)/day			
Mean (SD, *N*)	0.52 (0.27, 27)	0.46 (0.27, 27)	0.51 (0.23, 27)
Minimum–Maximum	0–1.2	0.1–1.0	0.19–1.0

### Feedback on the Snacktivity™ Intervention (Single-Items Questions)

Of those providing data in the Snacktivity™ group at follow-up, most reported the trial/intervention helped them to think more about the amount of physical activity they did (27/28, 96%) and the time they spent sitting each day (26/28; 93%). Similarly, 17/20 (85%) reported the Snacktivity™ consultation was useful, most liked the Snacktivity™ approach (19/28, 68%), with a further 6/28 (21%) reporting neutral views. Snacktivity™ was considered easier to achieve on non-working days rather than working days. The SnackApp was considered helpful in facilitating Snacktivity™ in 22/27 (81%). The Fitbit device with the bespoke Snacktivity™ watch face was considered helpful in supporting participation in Snacktivity™ in 21/27 (78%).

The most enjoyed activity snacks were taking the stairs instead of the lift/escalator, short bouts of gardening, walking up and down stairs, walking in local parks and parking the car further away and walking to destination. Activity snacks enjoyed the least were lunges whilst vacuuming the house, skipping, adding some extra weight to a rucksack while walking and squats at a desk. Activity snacks considered easiest to build into daily routines were walking up and down stairs, marching on the spot, using the stairs instead of a lift/escalator, walking whilst talking on the phone, and short bouts of gardening. The activity snacks mostly likely to be completed at work were using the stairs instead of the lift/escalator, walking whilst talking on the phone, walking meetings with colleagues and a brisk walk at lunchtime.

### Secondary Outcomes and Serious Adverse Events

Data collected at baseline and follow-up for other outcomes of interest are presented descriptively in Electronic Supplementary Material 2: Tables 2 and 3. No serious adverse events were reported.

## Discussion

Guidance for physical activity now recognises the positive contribution that participation in short bouts of physical activity can have for health, and that any amount of physical activity is better than none [4]. This change to the guidance has provided opportunities to develop and test interventions that promote short(er) bouts of physical activity, this being the aim of this study. Whilst the green criteria for recruitment was achieved, this was over a longer period than planned, indicating that additional strategies to facilitate recruitment are required by future research evaluating the Snacktivity™ intervention. Depending on which scenario and assumptions are applied, the intervention promoted Snacktivity™ to varying degrees. Data indicated that on average the Snacktivity™ intervention group completed 9–10 activity snacks per day. Participants were generally enthusiastic about the Snacktivity™ approach to promoting physical activity, supporting the need for further research to investigate the effectiveness of the intervention. Attrition was green to amber (depending on the criterion applied), indicating that future research testing Snacktivity™ approaches may benefit from further strategies to maximise retention and data completeness.

### Recruitment

This feasibility trial provided the opportunity to try out different recruitment strategies. We originally aimed to recruit 80 participants over 5 months via two recruitment routes: patients receiving an NHS Health Check in primary care and within community health consultations. A total of 72/80 participants were recruited (90% of target), over 14 months, longer than the original target of 5 months. At the time of this study, there were significant workforce challenges in primary care where the focus was on delivering the national COVID-19 vaccination programme, and consequently NHS Health Checks were not being routinely offered by practices to patients. We proactively discussed alternative recruitment options with the practices and adapted our recruitment model to include the option of delivering the Snacktivity™ intervention within other types of routine health consultations (e.g. immunisations, blood pressure checks). Whilst practices agreed to do this, it remained logistically challenging for them to accommodate the timelines required by the trial, whilst also dealing with the ongoing pressures of COVID-19. In the community health settings, we recruited from podiatry, dentistry, dietetics, and physiotherapy services. Many community healthcare providers were working remotely during COVID-19, redeployed to COVID-19 related work tasks and/or were not completing their usual work, all of which impacted on our ability to recruit from this setting. Despite challenges with recruitment in the trial, there was good engagement from HCPs with delivering the Snacktivity™ intervention to participants [36].

The trial data collection procedures also required a home visit to participants, and this may have deterred people from participating following the COVID-19 pandemic and may have been viewed as burdensome. In future research we plan to remove the need for a home visit as accelerometers for the collection of the primary outcome data (minutes of MVPA) can be posted to participants at baseline and follow-up. We hope this strategy will facilitate recruitment and make the trial more attractive to the public.

From the trial invitations sent directly to patients by health services, 1.3% were eventually randomised via this route. Whilst this rate was expected, it was over a longer period than anticipated. As the trial was experiencing difficulties with slow recruitment through the above two health services routes, recruitment through social media was included towards the end of the recruitment period. It was felt important to expand recruitment to include social media because the NHS is not the only context in which the Snacktivity™ approach could be offered to the public, if shown to be effective. We also wished to adopt a more inclusive approach to recruitment, rather than relying entirely on health services to identify potential participants. Recruiting via social media proved fruitful and likely to be useful in subsequent research. Nevertheless, additional strategies are required to facilitate recruitment in any subsequent phase III trial.

Another challenge around recruitment was related to the use of the GPPAQ to screen potential participants for inclusion [28]. We initially opted to use the GPPAQ because it has been used in primary care within NHS Health Checks. However, we found that it is not a good measure to screen for baseline physical activity levels in trials, with 40% of those interested in participating in this trial, and who might benefit from taking part, classified as ineligible by the GPPAQ. In many instances, this was because those interested reported that they had an apparently active job (e.g. plumber or car mechanic) and the GPPAQ would, by default, define these individuals as active and consequently ineligible to take part. In truth, many were likely to be eligible as they were doing little MVPA, or any additional activity outside of their occupation. Other studies have also highlighted difficulties with categorising participants as active and inactive using the GPPAQ and we propose using an alternative physical activity screening tool in future research [45].

### Adherence and Thoughts About Snacktivity™

Motivation is core to behaviour change, and for Snacktivity™ to be successful, it will be important to understand participant’s engagement with this approach within their lives. The data collected via the Fitbit device allowed us to understand how many activity snacks intervention participants completed each day/week. Regarding the stop–go criteria, the difference in the results between scenario 1 and scenario 2 was the result of missing data related to none or insufficient wear time of the Fitbit. There are several potential explanations. In scenario 2, 15 participants did not wear the Fitbit for sufficient time/days, leading to these participants being categorised as having ‘insufficient wear time’, and another five participants did not wear the Fitbit at all. We could speculate this may be because participants felt they did not need or wish to wear the Fitbit to complete their Snacktivity™ and/or because they had access to other Snacktivity™ intervention resources for support, such as the SnackApp™ on their phone, and the Snacktivity™ picture board. The study monitoring systems also identified that some participants would place their Fitbit to charge and then fail to put it back on their wrist, leading to some missing data impacting the stop–go results. We have learnt that we need to include more prompts to remind participants to put their Fitbit back on after charging, so that more complete data regarding Snacktivity™ behaviour can be collected. Consistent with the aims of the intervention, this may further facilitate participants’ self-monitoring of behaviour and help with habit formation for Snacktivity™ [29, 30]. This information may also be of benefit to other research that relies on data from consumer tracker devices to demonstrate the fidelity of physical activity interventions. It was nevertheless encouraging to see the intervention group were able to complete an average of 9–10 activity snacks per day.

An important driver of adherence to Snacktivity™ is likely to be individuals’ views about the approach. Consistent with previous research, overall, the intervention group was supportive of the approach [16, 21, 22, 35, 36]. Most of the intervention participants thought that the trial had helped them to think more about the amount of physical activity and time they spent sitting each day, pointing to self-regulation and self-reflection of behaviour. Regarding the intervention specifically, most participants thought the brief Snacktivity™ consultation was useful. Like other studies, Snacktivity™ was considered easier to achieve on non-working days than working days [16] and this information can be used to further refine the focus of the intervention. In line with self-regulation theory and the habit formation model, the SnackApp aims to help participants to monitor their participation in activity snacks and promote the development of habits for activity snacks throughout the day [29, 30]. The SnackApp™ was considered helpful in facilitating participation in Snacktivity™ and the Fitbit device was also reported to be helpful. These findings support previous reports detailing the development of the SnackApp™, as well as other studies reporting the potential usefulness of technology to support physical activity [31, 35, 46].

### Participation in MVPA

Most of the population are not sufficiently physically active [1, 2]. Snacktivity™ seeks to support people who are physically inactive to move towards achieving participation in at least 150 min of MVPA each week, which can be a difficult health behaviour to change and sustain. Snacktivity™ offers an opportunity to promote the view that physical activity can be a convenient, simple and achievable behaviour which can be completed at any time of the day, and in any environment. Whilst there were some missing data, the proportion of participants in the Snacktivity*™* group who accumulated a total weekly average of at least 105 min of MVPA (~ 15 min daily) met the amber stop–go criterion, demonstrating that the Snacktivity™ approach has the potential to move people from being inactive to completing modest amounts of MVPA per week. This is important because MVPA has an inverse dose–response relationship with all-cause mortality and even small amounts of physical activity can reduce the risk of all-cause mortality [47, 48].

### Strengths and Limitations

No prior RCT has explored the benefits of Snacktivity™ in promoting physical activity. We included the option of visits to participants’ homes for the collection of directly measured health data, rather than relying on self-reported data. This study focused on recruiting people who were predominately inactive at baseline, a population most in need of support and who are likely to benefit the most from intervention. Whilst recruitment took longer than expected, participants were recruited from a wide range of ethnic backgrounds including those with pre-existing diseases/conditions (e.g. hypertension, depression, overweight/obesity and back pain). From a behaviour change perspective, Snacktivity™ might be a more feasible and appealing alternative for those who are not currently able or willing to be physically active. Data completeness and attrition were acceptable but need improvement in the full trial. Additional prompts are required in the intervention to ensure participants wear the Fitbit device for at least 10 h each day and that they re-wear after charging the device. The next step for this work is to test the effectiveness and cost-effectiveness of the Snacktivity™ intervention, ensuring high follow-up rates at 3 and 12 months. Overall, the stop–go criteria findings from this feasibility trial indicate proceeding to such a trial, but with an internal pilot to provide an opportunity to address the issues identified in this feasibility trial.

## Conclusion

Novel interventions, such as Snacktivity™, that seek to support the public to increase their physical activity are required. Our findings can be used to inform the design of subsequent research that seeks to investigate whether innovative approaches such as Snacktivity™ are effective in promoting physical activity. If confirmed in future RCTs, these findings could inform future public health messaging that seeks to raise awareness to the population of the potential health benefits from Snacktivity™.

## Supplementary Information

Below is the link to the electronic supplementary material.

Supplementary file1 (PNG 756 KB)

Supplementary file2 (DOCX 26 KB)
