# Supplementary figures and images for: Snacktivity™ to Promote Physical Activity in Primary Care, Community Health and Public Health Settings: A Feasibility Randomised Controlled Trial
Source: Int J Behav Med. 2025 Feb 24;32(5):702–16. doi: 10.1007/s12529-025-10352-3 (PMC12672755; doi:10.1007/s12529-025-10352-3)

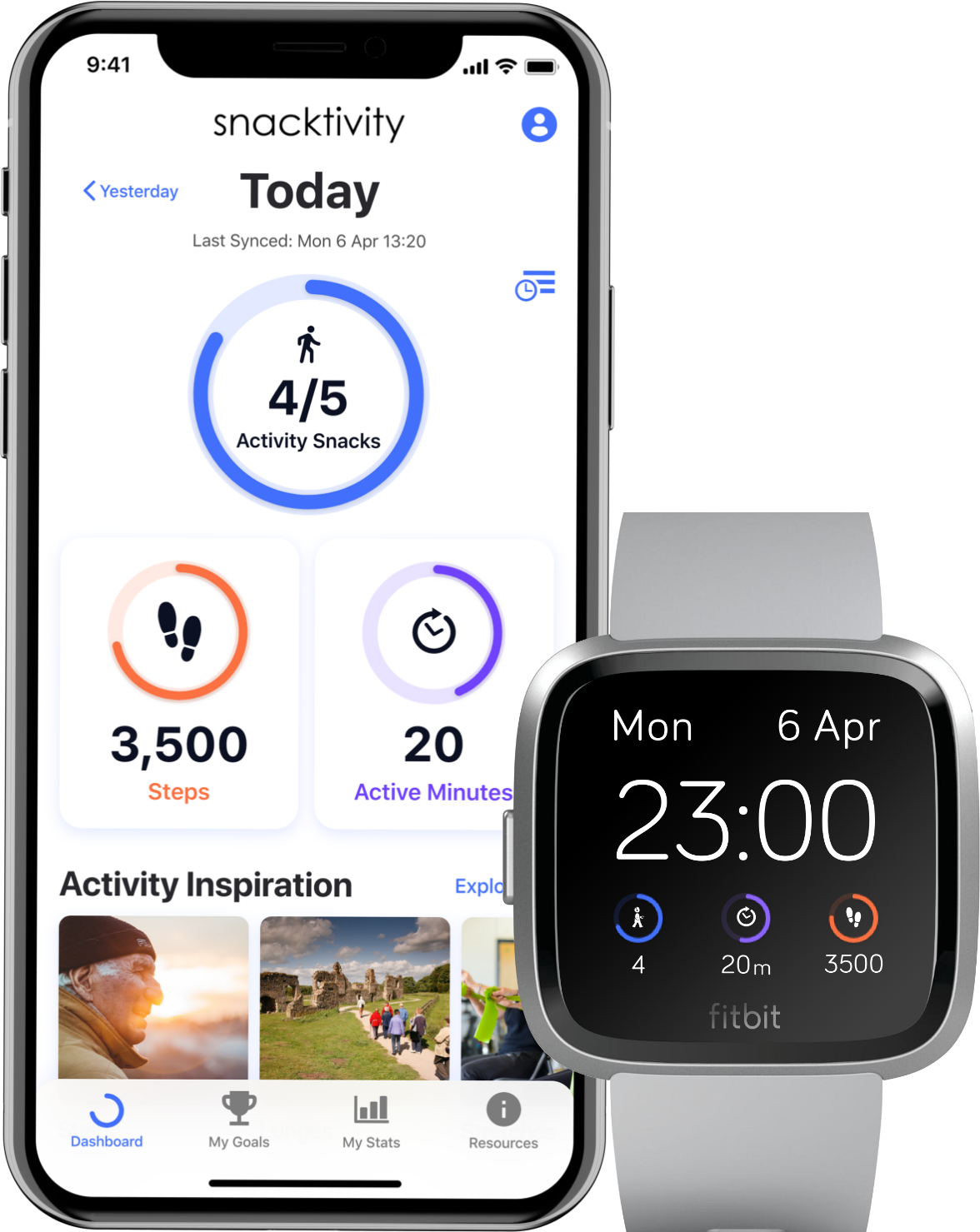

Supplement: Supplementary file 1 — Supplementary file1 (PNG 756 KB) [file 12529_2025_10352_MOESM1_ESM.png]
