# Supplementary material for: Snacktivity™ to Promote Physical Activity in Primary Care, Community Health and Public Health Settings: A Feasibility Randomised Controlled Trial
Source: Int J Behav Med. 2025 Feb 24;32(5):702–16. doi: 10.1007/s12529-025-10352-3 (PMC12672755; doi:10.1007/s12529-025-10352-3)
Supplement: Supplementary file 2 — Supplementary file2 (DOCX 26 KB) [file 12529_2025_10352_MOESM2_ESM.docx]

**Supplementary Table 1: Schedule of assessments**

| Visit | Screening | Baseline visit (-7 + 14 days) | Follow up  (12 weeks)  (- or + 14 days) |
| --- | --- | --- | --- |
| Expression of interest | x |  |  |
| Physical activity status: General Practice Physical Activity Questionnaire) (27) | x |  |  |
| Eligibility screening telephone call | x |  |  |
| Personal identifiers and demographic information |  | x |  |
| Current medications |  |  | x |
| Smoking history |  | x |  |
| Alcohol consumption |  | x |  |
| Mobility |  | x |  |
| Diseases/conditions |  | x | x |
| Wrist worn accelerometer (worn for up to 8 days) (Axivity) |  | x | x |
| Anxiety and Depression: Hospital Anxiety and Depression Scale (38) |  | x | x |
| Healthcare utilisation |  |  | x |
| Household income/composition |  |  | x |
| Productivity |  |  | x |
| Sedentary behaviours: Workforce Sitting Questionnaire (36) and sedentary behaviour item from the International Physical Activity Questionnaire (37) |  | x | x |
| Enjoyment of physical activity: Physical Activity Enjoyment Scale (39) |  | x | x |
| Self-efficacy for exercise and Exercise Self-efficacy Questionnaire (40) |  | x | x |
| Habit strength (Snacktivity*™* group): The Self-Report Habit Index (41) |  |  | x |
| SnackApp™ engagement analytics (Snacktivity*™* group) |  |  | x |
| Height* |  | x |  |
| Weight |  | x | x |
| Body mass index (BMI) |  | x | x |
| Waist circumference |  | x | x |
| Lower limb muscle strength (Takei dynamometer squat position) |  | x | x |
| Blood pressure |  | x | x |
| Checklist of popular snacks (paper copy, Snacktivity*™* group only) |  |  | x |
| Semi-structured interviews (Snacktivity*™* group and health care providers) |  | x | x |
| Single item study feedback questions |  |  | x |

* To allow for the calculation of BMI, height was be measured to the nearest 0.1 cm using SECA 213 stadiometers at baseline and follow up.

**Supplementary Table 2: Secondary health outcomes by treatment group**

|  | **Baseline** | | **12 week follow-up** | | Mean difference^1^ (95% CI) |
| --- | --- | --- | --- | --- | --- |
|  | Snacktivity™ | Usual care | Snacktivity™ | Usual care |  |
| Lower limb muscle strength (kg) | | | | | |
| Mean (SD, N) | 48.1 (23.9, 27) | 45.5 (19.6, 28) | 58.0 (23.0, 23) | 55.2 (25.3, 26) | -0.7  (-11.6 to 10.2) |
| Missing | 10 | 7 | 14 | 9 |  |
| Weight (kg) | | | | | |
| Mean (SD, N) | 81.4 (20.9, 37) | 83.4 (27.6, 35) | 84.5 (24.8, 30) | 81.0 (29.3, 30) | 2.1 (-3.0 to 7.2) |
| Missing | 0 | 0 | 7 | 5 |  |
| BMI (kg/m^2^) | | | | | |
| Mean (SD, N) | 29.4 (7.2, 37) | 31.2 (9.5, 35) | 29.9 (9.4, 30) | 30.6 (10.3, 30) | 1.0 (-1.0 to 2.9) |
| Missing | 0 | 0 | 7 | 5 |  |
| Waist circumference (cm) | | | | | |
| Mean (SD, N) | 97.3 (16.4, 37) | 98.8 (15.7, 35) | 96.6 (15.7, 30) | 96.6 (18.5, 30) | -1.5 (-4.2 to 1.1) |
| Missing | 0 | 0 | 7 | 5 |  |
| Systolic blood pressure (mmHg) | | | | | |
| Mean (SD, N) | 124.4 (18.6, 37) | 129.0 (20.4, 34) | 124.2 (15.1, 30) | 125.6 (19.1, 30) | 2.0 (-4.2 to 8.3) |
| Missing | 0 | 1 | 7 | 5 |  |
| Diastolic blood pressure (mmHg) | | | | | |
| Mean (SD, N) | 77.9 (9.8, 37) | 78.8 (11.3, 34) | 78.9 (7.6, 30) | 78.5 (10.0, 29) | 1.7 (-2.2 to 5.6) |
| Missing | 0 | 1 | 7 | 6 |  |
| Hospital Anxiety and Depression Scale anxiety score* | | | | | |
| Mean (SD, N) | 7.5 (5.1, 37) | 7.3 (4.5, 35) | 6.5 (4.6, 28) | 6.9 (4.7, 28) | -0.9 (-2.4 to 0.7) |
| Missing | 0 | 0 | 9 | 7 |  |
| Hospital Anxiety and Depression Scale depression score* | | | | | |
| Mean (SD, N) | 6.1 (4.4, 37) | 6.4 (4.3, 35) | 4.5 (3.8, 28) | 4.9 (4.3, 28) | -0.2 (-1.6 to 1.1) |
| Missing | 0 | 0 | 9 | 7 |  |
| International Physical Activity Questionnaire total sitting time (minutes/day) | | | | | |
| Median [IQR, N] | 385.7 [300.0-535.7, 37] | 424.3 [338.6-552.9, 35] | 317.1 [265.7-487.6, 27] | 409.3 [314.6, 28] | -92.1^2^ (-199.7 to 15.4) |
| Missing | 0 | 0 | 10 | 7 |  |
| Workforce Sitting Questionnaire total sitting time (minutes/day) | | | | | |
| Median [IQR, N] | 670.7 [457.5-803.6, 36] | 607.9 [460.0-860.0, 35] | 600.0 [360.0-801.4, 27] | 657.9 [512.1-823.6, 28] | - |
| Missing | 1 | 0 | 10 | 7 |  |

Abbreviations: BMI=Body Mass Index cm=centimetres, CI=Confidence Interval, IQR=interquartile range, kg=kilograms, m=metres, mmHg=millimetres of mercury, N=Number of observations, SD=Standard deviation.

^1^Adjusted for minimisation variables (recruiting service/route, gender and age) and baseline value. Values <0 indicate lower limb muscle strength/lower weight/lower BMI/lower waist circumference/lower systolic and diastolic blood pressure/lower anxiety and depression scores in Snacktivity™.

^2^Unadjusted difference in medians. Values <0 indicate less sitting time in Snacktivity™.

^*^HADS domain scores range from 0 to 21, where higher scores are bad.

**Supplementary Table 3: Accelerometery data by treatment group**

|  | **Baseline** | | **12 week follow-up** | | Mean difference (95% CI) at follow-up adjusted for baseline & other minimisation variables |
| --- | --- | --- | --- | --- | --- |
|  | Snacktivity™ (N=37) | Usual care (N=35) | Snacktivity™ (N=37) | Usual care (N=35) |  |
| Number of minutes/day of MVPA | | | | | |
| Mean (SD, N) | 37.5 (25.3, 31) | 27.5 (23.0, 33) | 42.9 (33.3, 24) | 45.6 (97.6, 23) | -11.6^1^ (-63.4 to 40.2) |
| Number of minutes/day of MVPA (sensitivity analysis^4^) | | | | | |
| Mean (SD, N) | 37.5 (25.3, 31) | 27.9 (23.3, 32) | 42.9 (33.3, 24) | 25.8 (21.9, 22) | 4.8^1^ (-11.5 to 21.2) |
| Number of minutes/day of light physical activity | | | | | |
| Mean (SD, N) | 233.9 (64.9, 31) | 243.3 (61.8, 33) | 279.7 (109.9, 24) | 300.0 (129.4, 23) | -7.4^1^ (-80.9 to 66.1) |
| Number of minutes/day of total physical activity | | | | | |
| Mean (SD, N) | 313.5 (78.8, 31) | 314.1 (89.8, 33) | 362.8 (120.3, 24) | 389.7 (196.8, 23) | -23.6^1^ (-127.2 to 80.1) |
| Average acceleration mg/day | | | | | |
| Mean (SD, N) | 23.3 (5.6, 31) | 22.5 (6.8, 33) | 27.5 (7.7, 24) | 29.1 (15.9, 23) | -2.2^1^ (-10.3 to 5.9) |
| Number of minutes/day of sedentary time | | | | | |
| Mean (SD, N) | 683.6 (87.9, 31) | 667.9 (99.6, 33) | 630.9 (121.2, 24) | 597.5 (188.9, 23) | 16.8^2^ (-83.1 to 116.6) |
| Number of minutes/day of sleep time | | | | | |
| Mean (SD, N) | 442.9 (56.0, 31) | 458.0 (76.4, 33) | 446.3 (49.5, 24) | 452.7 (65.3, 23) | 4.4^3^ (-29.5 to 38.3) |

Abbreviations: CI=Confidence Interval, IQR=Interquartile Range, mg=Milligram, MVPA=Moderate-vigorous physical activity, N=Number of observations, SD=Standard deviation.

Note: All available data presented include participants with ≥1 valid wear day..

^1^Adjusted for minimisation variables (recruiting service/route, gender and age) and baseline value. Values >0 favour Snacktivity™.

^2^Adjusted for minimisation variables (recruiting service/route, gender and age) and baseline value. Values <0 favour Snacktivity™.

^3^Adjusted for minimisation variables (recruiting service/route, gender and age) and baseline value. Values <0 indicate lower sleep time in Snacktivity™ group.

^4^Sensitivty analysis where participant who is an outlier removed from baseline and follow-up (482.4 minutes of MVPA/day at follow-up in usual care arm).
